# Apoptosis Induced by Prednisolone Occurs without Altering the Promoter Methylation of *BAX* and *BCL-2* Genes in Acute Lymphoblastic Leukemia Cells CCRF-CEM

**DOI:** 10.31557/APJCP.2020.21.2.523

**Published:** 2020

**Authors:** Saiedeh Ganbarjeddi, Ako Azimi, Milad Zadi Heydarabad, Maryam Hemmatzadeh, Shahin Mohammadi, Reza Mousavi Ardehaie, Majid Zamani, Sina Baharaghdam, Sajjad Esmaeili, Amin Ghasemi

**Affiliations:** 1 *School of Medicine, Karazin Kharkiv National University, Kharkiv, Ukraine, *; 2 *Department of Basic Science, *; 7 *Student Research Committee, Maragheh University of Medical Sciences, Maragheh, *; 3 *Medicinal Plants Research Center, Yasuj University of Medical Sciences, Yasuj, *; 4 *Department of Immunology, School of Medicine, *; 5 *Student Research Committee, *; 6 *Immunology Research Center, Tabriz University of Medical Sciences, Tabriz, Iran. *

**Keywords:** Prednisolone- DNA methylation- acute lymphoblastic leukemia- apoptosis- methylation specific PCR

## Abstract

**Objective:**

one of the main mechanisms in which cancer cells are resistant to chemotherapy drugs and therapeutic strategies is resistance to apoptosis due to these anticancer factors. Regulating the expression of genes through epigenetics, especially regulation through methylation, is one of the key aspects of regulating gene expression and the function of genes, which is also regulated by the pathways regulating the pathway of apoptosis. The epigenetic regulatory phenomenon in cancer cells can undergo a change in regulation and induces resistance to apoptosis against chemotherapy and anticancer factors. The purpose of the present scrutiny was defined to probe the effect of subtoxic prednisolone dose on the level of promoter methylation and gene expression of *BAX* and *BCL2 *in the CCRF-CEM cells.

**Methods:**

The treated cells by prednisolone, cultured in RPMI 1640 medium in standard condition. Alteration in promoter DNA methylation was analyzed by use of methylation specific-PCR (MSP) technique after the defined intervened time of Prednisolone treatment with a subtoxic dose.

**Results:**

Prednisolone can induce apoptosis via alteration in *BAX* and *BCL2* genes, based on our previous scrutiny. This essay shows no varies in the Pattern of DNA methylation of examined genes; however, prednisolone changes the expression of examined genes.

**Conclusion:**

Lack of alteration through prednisolone treatment in DNA methylation template of *BAX* and *BCL2* genes make this possible that Prednisolone affects apoptotic gene expression via different pathways, which need more research to be done about it.

## Introduction

Acute Lymphoblastic Leukemia (ALL) is the most common type of childhood leukemia, which is generally recognized in marrow and blood through an acute attack and rapid aggregation of immature leukemia cells (blast) with a common peak at the age of 2-5 years (Holleman et al., 2004; Azimi et al., 2016). The outbreak of ALL in the world is roughly 5 per 100,000 populations per year (Estey and Döhner, 2006; Yousefi et al., 2016; Ahani-Nahayati et al., 2018). ALL patients, despite chemotherapy drugs, usually suffer from the side effects of these chemotherapy drugs and the risk of recurrence of the disease (Zadi Heydarabad et al., 2018). Apoptosis is a planned death that usually takes place to harmonize a balance between production and cell death of cells even in normal cells and is needed to maintain cellular homeostasis, a regulatory disorder that causes this disease including cancer (Hervouet et al., 2013). On one hand, one of the principal pathways which make malignant cells resistant to anticancer therapies and therapeutic strategies is resistance to apoptosis. BCL2 family members act as a critical regulator of mitochondrial pathway of apoptosis which potentially consider as target in leukemia therapy. Furthermore, one of the important approach in this regard is down regulation of anti-apoptotic members such as BCL2 protein and on the contrary induction of pro survival members embracing BAX protein. One of the crucial approach to alter the gene expression is epigenetic. DNA methylation of promotor in GpC island consider as common mechanism for inhibiting the gene expression in cancerous cells (Vahdani et al., 2019; Hervouet et al., 2013). This resistance to cancer cell apoptosis is usually one of the scientists’ major challenges in overcoming cancerous cells. Therefore, with full knowledge of the mechanisms of resistance, it is possible to overcome this drug resistance of cancer cells and can be an important step in improving the condition of these patients. Furthermore, full recognition of the mechanism of drug resistance to apoptosis is necessary to design new therapies to eliminate cancer cells. On the other hand, the regulation of gene expression through epigenetics, especially regulation through methylation, is one of the key aspects of regulation of gene expression and the function of genes, which is also applied to regulator genes of the apoptosis’ pathway. The phenomenon of epigenetic regulation in cancer cells can undergo a change in regulation and induces resistance to apoptosis against chemotherapy and anticancer agents (Hervouet et al., 2013; Zadi Heydarabad et al., 2018). Moreover, the development of analytical tools and extensive analysis of the genome emphasized a great deal on the prevalence of epigenetic changes in cancer. Therefore, an accurate perception of the mechanisms which accompanied with resistance to Glucocorticoid drugs in leukemic patients, including a comprehensive understanding of epigenetic changes, can be a new strategy to overcome resistance to GC and improvement of outcomes (Bachmann et al., 2010). 

The aim of the current research was defined to probe the effect of subtoxic prednisolone dose on the level of promoter methylation and gene expression of *BAX* and *BCL2* in the CCRF-CEM cells. Glucocorticoid (GC) is a class of stress-induced steroid hormones that regulate various immunological, metabolic, homeostatic and cardiovascular functions (Pelt, 2010). One of the synthetic glucocorticoids used in ALL disease is prednisolone. Prednisolone is often used to suppress the immune response and also to cure the inflammatory diseases and autoimmune diseases including Chronic obstructive pulmonary disease (COPD), asthma, Crohn’s disease, multiple sclerosis, sarcoidosis, blood cancers, such as ALL, multiple myeloma, and non-Hodgkin (Pickup, 1979; Tiso et al., 2004). Glucocorticoids bind to glucocorticoid receptors for their function and eventually inhibit the immune system by altering gene expression. Common side effects of prednisolone include fatigue, increased blood pressure, and blood glucose, mental changes, headaches, and long-term use may cause cataracts (Louis, 2011). GC induces activation of caspase cascade and induces apoptosis in the cell line of lymphoblastic leukemia by increasing in BAX protein and decreasing the BCL2 protein (Azimi et al., 2015; Ghasemi et al., 2018). We know that the level of methylation of the promoters of most genes is associated with their expression, so that the reduction of methylation or hypomethylation triggers the activation of the genes and the increase of methylation inhibits these genes (Heydarabad et al., 2016; Marofi et al., 2019). On one hand, hypomethylation of the oncogenes and the hypermethylation of tumor suppressor genes (TSGs) can lead to disease and cancer (Garcia-Manero et al., 2002; Hervouet et al., 2013). Therefore, regulating the expression of genes through epigenetics, especially regulation through methylation, is one of the key aspects of regulating gene expression and gene function, and this regulatory phenomenon applies to regulator genes of apoptosis’ pathway. In other words, epigenetic modifications, especially methylation, can contribute to the balance between the expression of pre-apoptotic and anti-apoptotic proteins, which this epigenetic regulatory phenomenon in cancer cells can undergo a regulatory change and induce resistance to apoptosis against chemotherapy and anti-cancerous factors (Hervouet et al., 2013). This change in the pattern of methylation has been reported in some cancers, for example, previous reports have shown that in glioblastoma, the increase in BAX promoter methylation induces a severe reduction in its expression (Cartron et al., 2002; Hervouet et al., 2010). Also in prostate cancer, the same increase in methylation is reported for *BAK* and *BIK *genes (Hervouet et al., 2010). Since prednisolone, by changing the expression of *BCL-2* family of proteins, induces apoptosis in leukemia cells (Ghasemi et al., 2018), the induction of apoptosis and changes in the expression of *BAX* pre-apoptotic genes and anti-apoptosis* BCL2* by changing methylation levels of Transcription Start Cite, we have tried, in this study, to find out whether the promoter of these genes occurs by reducing the methylation (hypo) of the *BAX* gene and increasing the methylation of the *BCL2 *gene or not. For this purpose, in this study, we have selected the CCRF-CEM cell line as a representative of human ALL to study the promoter’s methylation changes following drug treatment in a time-dependent manner. A complete understanding of the naturally occurring drug kinetics in leukemia treatment would contribute to a clear view of the cure mechanisms and can pave the way towards designing safer drugs that can target specific pathways which are discouraged during leukemogenesis. The aim of the current research was defined to probe the effect of sub-toxic prednisolone dose on the level of promoter methylation and gene expression of *BAX* and *BCL2* in the T acute lymphoblastic leukemia cell line (CCRF-CEM).

## Materials and Methods

*Cell Culture*

CCRF-CEM as a model of the leukemic cells was obtained from National Cell Bank (Pasteur Institute of Tehran, Iran) and maintained in RPMI 1640 medium, containing 10% fetal calf serum (GIBCO, USA), in a humidified atmosphere with 5% CO_2_ at 37^o^C. The CCRF-CEM cells (4 ×10^5^ cell/ml) were plated in 60mm culture plate for treatment with a single dose of prednisolone.

*Treatment*

Prednisolone (98 % purity, Sigma-Aldrich, Germany) was dissolved in dimethylsulfoxide and diluted in RPMI 1640 medium to a suitable concentration. The appropriated dose of prednisolone (700 µM) added to CCRF-CEM cells. After 24 and 48 h, treated cells prepared for extraction of DNA and protein (Khanzadeh et al., 2018).

*Genomic DNA Extraction and Sodium Bisulfite Modification*

The genomic DNA was isolated from CCRF-CEM cells by Inc. FlexiGene DNA kit (Qiagen, Germany) based on the manufacturer instructions. DNA concentration and purity were evaluated by spectrophotometer in 260 and 280 nm wavelengths. DNA integrity was determined via electrophoresis in 1% agarose gel for the next steps which are treatment by sodium bisulfite. 

Treatment of approved genomic DNA templates by sodium bisulfite was done for transmutation of unmethylated cytosine to uracil. Incubation of 1.5 µg DNA was performed for 20 minutes at 37^o^C for primary denaturation with 0.2 M NaOH. Then in a volume of 500 µL, treatment by hydroquinone and sodium bisulfite (pH=5) at the dose of 10 mM and 3.5 M, were done, respectively. After that treatment condition changed and treated cells incubated beneath of mineral oil at 56^o^C for 16 hours. The clean-up columns (Qiagen, Germany) were appropriated according to the instructions for purifying the transmuted DNA. Elution buffer in a volume of 150 µL was used to elute the treated cells.

NaOH in the concentration of 0.3 M was used to desulfonate the eluted treated cells and then samples incubated for 5 minutes at the room temperature. For neutralization of solution, Ammonium acetate (pH=7.0) in the concentration of 3M was added. After that, 4 volumes of ethanol were used to precipitate the DNA and then the pellet of DNA was resuspended in 25 µL of distilled water. Transmuted DNA with Bisulfite were appropriated for MSP step or stored at -20^o^C for next evaluation. 

*SssI methylase treatment and primer design*

Purified DNA treated by Sss1 methylase (Biolabs, New England, US) based on manufacturer’s instruction. The DNA which was treated by prednisolone applied as a positive control MSP technique. Specific primers for methylated and unmethylated CpG sites were designed using MethPrimer design tool (http://www.urogene.org/methprimer/). The primers were selected based on the capability to recognize the pattern of methylation on BAX and BCL2 promotor districts accompanied with transcriptional beginning zones. The properties of primers which were applied in this study are mentioned below in Table 1. 

*Methylation Specific PCR (MSP)*

MSP experience were done in an amount of 25µl containing bisulfite treated DNA (100 ng), specific primers (0.75 µM for each one), MgCl_2_ (2 mM), dNTP (0.2 mM), KCl (100 mM), Tris-HCl (pH 7.5, 20 mM), and AmpliTaq Gold (0.5 unit, Roche Molecular Systems Inc., Branchburg, New Jersey, USA). Thermal status of PCR test was elucidated including: primary denaturation at 95°C for 5 minutes and repeated among 40 cycles which last 40 seconds for each one, annealing temperature (30 seconds), 72°C (40 seconds), and a final 7 minutes for extension at 72°C using PTC-100 Programmable Thermal Controller (MJ Research Inc., Waltham, Massachusetts, USA). Electrophoresis in 2% agarose was applied to visualize the PCR product.

*Proteins extraction and separation*

The treated cells harvested and washed for protein extraction by cold phosphate saline buffer. The cell pellet was lysed with an equivalent amount of cell lysis buffer. This buffer contained 0.5% SDS, 1% NP-40, 10 mM Tris-HCl (pH=7.4), 0.5% sodium deoxycholate, 150 mM NaCl, 5 mM EDTA, 100 µM PMSF, an inhibitor of protease-phosphatase. The products were centrifuged for 20 min at 13,000 rpm in -4^o^C. Then supernatants were removed and the concentration of purified protein was evaluated by means of the Bradford method. According to Laemmli’s assay, the separation of the same amount of proteins was done by means of 10% SDS-PAGE.

*Western Blot*

The formed bands of the extracted protein were conveyed to the nitrocellulose membrane by means of mini Trans-Blot Bio-Rad. Washing the membrane and exposing to the blocking agent (5% skim milk in TBS-T, for 1 hour) were performed. TBS-T was used for washing the membrane and then encountered with primary monoclonal antibody (BAX: mouse IgG11, sc-7382, 1:1,000; BCL-2: mouse IgG2b, sc-7480, 1:1,000, Santa Cruz, California, USA) and blocking solution, simultaneously, for 24 h at 4^o^C. For evaluating the intensity of band formation, the anti-actin antibody was applied as an internal control. After three times of washing the membrane by means of TBS-T, it was exposed to anti-mouse secondary antibody (1:1,000, citomatin gene) which was conjugated with HRP for one hour at room temperature. Finally formed bands by means of chemiluminescence assay were visualized. It could be better to consider that western blot technique was done in triplicate for these proteins. All reactions were done in triplicate.

*Statistically Analysis*

The density of obtained bands were measured by ImageJ software. The data were analyzed by SPSS 20.0 software. The paired T-test was used for evaluating the variation between groups and p values less than 0.05 considered as statistically significant.

## Results

*Prednisolone treatment is ineffective on BAX and BCL-2 methylation in CCRF-CEM cells*

**Figure 1:**
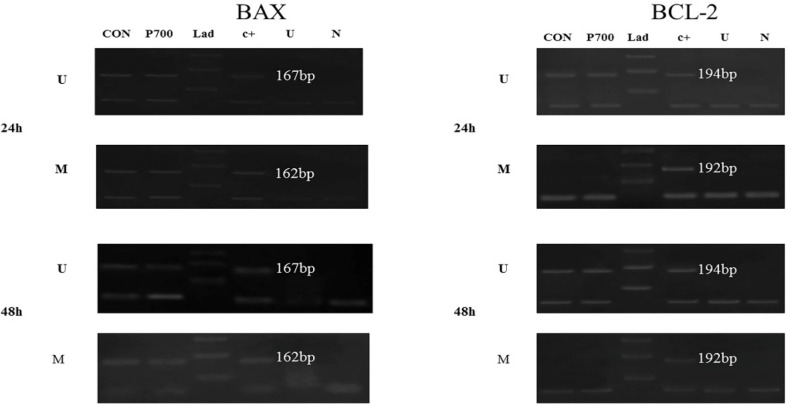
BAX and BCL2 Promoter Methylation by MS-PCR at 24 and 48 hours after Treatment with Perdnisolone. The results showed that the methylation pattern of *BAX* and *BCL-2* genes after treatment with Prednisolone 700μM were not changed. MSP amplified products with both methylated and unmethylated primers showed a partial methylation for *BAX* and *BCL-2* gene. Universally positive control for methylated and unmethylated DNA; P700, Prednisolone 700μM; L, Molecular marker; C+, Positive control; U, Unmethylated DNA; N, Negative control of PCR

According to the previous study, the dose of Prednisolone which was required to induce apoptosis in CCRF-CEM cell line is 700 µM. It has also shown, Prednisolone promotes the upregulation of *BAX* and downregulation of *BCL2* genes expression. Furthermore, the time had an important role in its effect. Thus, that seems very engrossing to design scrutiny for evaluating the promoter methylation profile related to how prednisolone may alter the *BA*X and *BCL2* gene expression. In order to probe the changes on the promoter methylation profile of target genes by prednisolone, the MSP technique was applied after 24 and 48 h incubation and then the outcomes were evaluated in comparison with untreated cells. According to this principle which treatment with bisulfate can change un-methylated cytosine, however, methylated one does not change, the primers were designed based on this principle which both of methylated and un-methylated sequences were targeted in promotor of *BAX* and *BCL2 *genes. The amplifications were done by thermocycler and the formed bands were remark as a methylation pattern of targeted genes. The results of amplification on both methylated and un-methylated primer pairs with or without PCR production consider as a CpG island methylation pattern at the start site of transcription (Figure 1). The results which were seen in Figure 1 illustrate prednisolone does not change the status of promoter methylation on *BAX* and *BCL2* genes. 

*Prednisolone up-regulates BAX while down-regulates BCL2 protein in CCRF-CEM*

**Figure 2:**
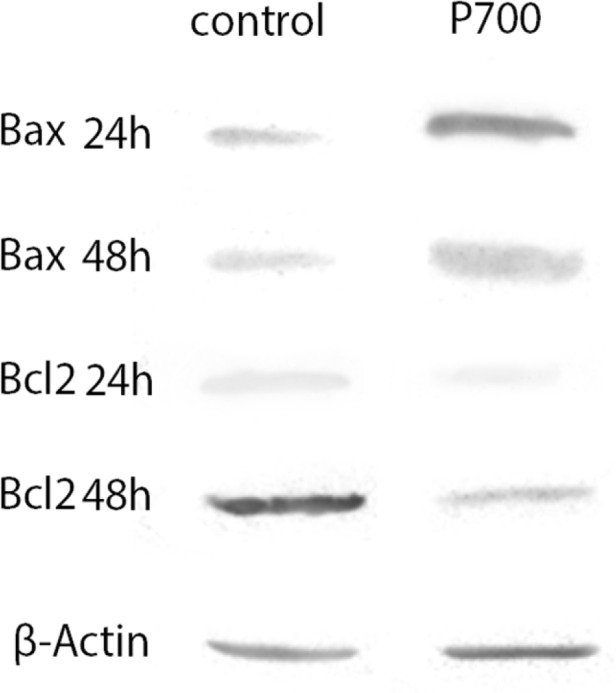
BAX and BCL-2 Protein Analysis by Western Blotting at 24 and 48 hours after Treatment with Prednisolone 700 μM. The BAX showed an intense band while BCL-2 proteins were minimally expressed in Prednisolone treated cells compared with non-treated cells

**Figure 3:**
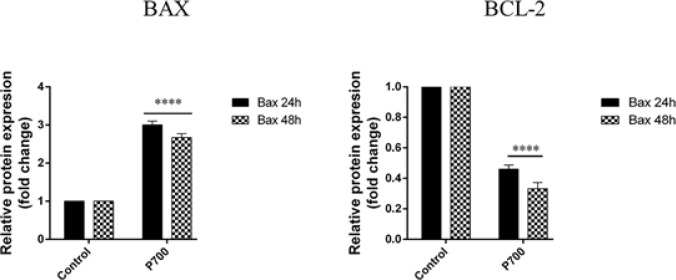
The BAX and BCL-2 Proteins Level. Results are representative of the increase in BAX protein and reduction in BCL-2 at doses of Prednisolone 700μM, after treatment, as compared to the protein level in control group (untreated cells). The band intensity of BCL-2 and BAX was quantitated and normalized with b-actin band and expressed as relative BCL-2 and BAX expression

The results of western blot showed that BAX was upregulated in prednisolone affected CCRF cells. Furthermore, after 48 h the BAX was higher than 24 h, it suggesting that prednisolone acts in a time-dependent manner. On the contrary, our data illustrated which BCL2 protein was decreased in a time-dependent manner after prednisolone treatment, as it clear in Figure 2. All in all, our scrutiny showed which treatment by prednisolone predisposed the CCRF cells to apoptosis induction via BAX upregulation and BCL2 downregulation. Moreover, the western blot outcomes analysis were illustrated in Figure 3 which show a decrease in BCL2 protein and an increase in BAX protein after 24 and 48 hours.

**Table 1:** Primer Characteristics used to Amplify the Promoter Regions of BAX and BCL2 Transcription Start Sites with CpG Rich Sequences

Primer	Sequence (5′—3′)	Tm (°C)	Product size (bp)	Amplified Region٭
BAX-MF	GTATTAGAGTTGCGATTGGACGG	59	162	48954657-489548819
BAX-MR	AAAATAACCGCTACCCCGC			
BAX-UF	GAAGGTATTAGAGTTGTGATTGGATG	58.5	167	48954653-489548820
BAX-UR	CAAAATAACCACTACCCCACAA			
BCL2-MF	GTTTTTAGCGTTCGGTATCGG	60	192	63319549-633197741
BCL2-MR	AAATCTCTATCCACGAAACCGC			
BCL2-UF	GGGTTTTTAGTGTTTGGTATTGG	59	194	63319549-633197741
BCL2-UR	AAATCTCTATCCACAAAACCACTTC			

*MF, Methylated Forward; MR, Methylated Reverse; UF, Unmethylated Forward; UR, Unmethylated Reverse. ٭ Nucleotide Number

## Discussion

Reports have shown that cancer is attributed predominantly to lifestyle, obesity, alcohol consumption, outdoor pollution, food additives as well as a small percentage of undifferentiated genes (Khan et al., 2010). One of the main mechanisms that cause cancer cells to become resistant to chemotherapy drugs and therapeutic strategies, is resistance to apoptosis due to these anticancer factors (Hervouet et al., 2013). This resistance to cancer cell apoptosis is usually one of the major challenges that scientists face in overcoming cancerous cells. Therefore, with full knowledge of the mechanisms of drug resistance, it is possible to overcome this drug resistance of cancer cells and can be an important step in improving the condition of these patients. Therefore, full recognition of the mechanism of drug resistance to apoptosis is necessary to design new therapies against cancer cells. On one hand, the regulation of gene expression through epigenetics, especially regulation through methylation, is one of the key aspects of regulating gene expression and the function of genes, which is also regulated by the pathways regulating the pathway of apoptosis (Bachmann et al., 2010). This phenomenon of epigenetic regulation in cancer cells can undergo a change in regulation On the other hand, the wide development of genomes’ analysis tools emphasized the prevalence of epigenetic changes in cancer. Therefore, the accurate perception about the mechanism of GC resistance in a patient involved ALL, including a comprehensive understanding of epigenetic changes, can be a new strategy to overcome GC resistance and improve outcome (Bachmann et al., 2010). And induce resistance to apoptosis against chemotherapy and anticancer factors (Bachmann et al., 2010). 

This study defined to probe the apoptotic effect of sub-toxic dose of prednisolone on the expression and level of promoter methylation of* BAX* and *BCL2* genes in CCRF-CEM acute lymphoblastic leukemia cells. Glucocorticoid (GC) is a class of stress-induced steroid hormones that regulate various immunological, metabolic, homeostatic, and cardiovascular functions (Smith and Cidlowski, 2010). One of the synthetic glucocorticoids used in ALL disease is prednisolone. Prednisolone is often used to control the immune system and also to treat inflammatory diseases and autoimmune diseases such as COPD, asthma, Crohn’s disease, multiple sclerosis, sarcoidosis, blood cancers, such as ALL, Multiple myeloma and lymphoma, etc. Hodgkin (Pickup, 1979; Tiso et al., 2004). Glucocorticoids bind to glucocorticoid receptors for their function and eventually inhibit the immune system by altering gene expression. Common side effects of prednisolone include fatigue, increased blood pressure and glucose, mental changes, headaches, and long-term use of it lead to cataracts, trash, bone loss (Buttgereit and Gibofsky, 2013). In the study by Lindsay Smith et al. They have found that GC has a wide range of physiological effects, including induction of apoptosis in lymphocytic malignancies. GCs usually have their physiological effects through binding to the glucocorticoid receptor, which, following this binding, transmits the receptor to the nucleus and ultimately activates or suppresses transcription of the glucocorticoid response genes. Due to the existence of a distorted distribution of GC and cognate receptors in the body, GC signalling results in a variety of physiological functions. For example, GC in the liver and adipose tissue causes positive regulation of the metabolism, which increases gluconeogenesis and lipolysis. Although GC in the immune system causes its inhibition by inducing abnormal apoptosis in the cell cycle and inflammation inhibition is caused by inhibition of inflammatory cytokines (Smith and Cidlowski, 2010; Papich, 2015). Researches have shown that GC affects cell proliferation and cell survival, differentiation, and metabolism in many tissues. GC in the lymphoid system produces IG, progresses the cell cycle and induces apoptosis in immature lymphoblast (Ghasemi et al., 2018; Khanzadeh et al., 2018). Studies have also shown that GC regulates immune responses and is clinically used to treat lymphoid malignancies, including ALL (Bachmann et al., 2010; Pelt, 2010; Smith and Cidlowski, 2010; Papich, 2015; Azimi et al., 2016). By inducing pre-apoptotic BCL-2 proteins, including BAX proteins, GC exacerbates the expression of the anti-apoptotic BCL-2 protein by causing caspase cascade and inducing apoptosis in the cell line of lymphoblastic leukaemia (Ghasemi et al., 2018). Adjusting gene expression through epigenetics, especially regulation through methylation, is one of the key aspects of regulation of gene expression and gene operation, and this regulatory phenomenon is also true for regulator genes of apoptosis’ pathway (Azimi et al., 2015; Zadi Heydarabad et al., 2018). In other words, epigenetic modifications, especially methylation, can contribute to the balance between the expression of pre-apoptotic and anti-apoptotic proteins, which this epigenetic regulatory phenomenon in cancer cells can undergo a regulatory change and induce resistance to apoptosis against chemotherapy and anticancer factors (Garcia-Manero et al., 2002). In a study conducted by Jones et al., (2017) On AAV patients (ANCA-associated vasculitis) evaluated the role of prednisolone on the DNA methylation pattern on active and inactive samples and showed that median methylation in PRTN3 promoter is independent of GC treatment and is active in patients suffering from the disease. Their study showed that DNA hypomethylation is independent of GC therapy without considering time on the treatment. The results also showed that the level of methylation of LFT, *ELANE* and *CGI* genes was low and there were no discrepancies in comparison with the control group in different conditions. However, the DNA methylation level for *MPO *and *PRTN3* genes induces in patients with active AAV compared to patients undergoing remission (Jones et al., 2017). Their study, regardless of the limitation on total leukocytes containing different percentages of different cell types among different types of blood cells, indicated that GC had no effect on the methylation of these genes (Jones et al., 2017). Zhang et al., (2015) by studying Histone deacetylases (HDACs) in ALL children, showed that *HDAC1*, *HDAC2*, and *HDAC8 *expression were significantly higher in all samples. Their results also indicated that high expression of *HDAC4* was associated with high numbers of primary leukocytes, ALL, T cells and poor response to prednisolone. They stated in their study that HDAC4 could be a drug target in ALL patients. In this study, for the first time, changes in the level of methylation of* BAX* and *BCL2 *genes induced by prednisolone in the CCRF-CEM chemotherapy-resistant cell line have been evaluated. One of the limitations of using GCs is the lack of a precise mechanism of cellular resistance to the drug, which remains unclear (Garcia-Manero et al., 2002; Hervouet et al., 2013). Bachmann et al., (2010) showed that resistance to glucocorticoids in children with ALL can be due to epigenetic changes caused by the extinction of the *BIM *gene. Patients with ALL GI Xenografts Resistance to GC have always failed in enhancing the expression of *BIM *(increased regulatory expression of *BIM *gene) after exposure to dexamethasone despite the presence of a functional GC receptor (Garcia-Manero et al., 2002; Hervouet et al., 2013). Although there were no overall changes in the methylation level of the CpG islands in the *BIM* gene, their results showed that GC resistance in xenograft and biopsy patients was significantly associated with a decrease in histone-3 acetylation. They also showed that vorinostat, a histone deacetylases inhibitor, reduced the suppression of* BIM* expression, as well as synergistic anti-cancer effects with dexamethasone both in vitro and in vivo levels (Hervouet et al., 2013). These kinds of data made us design scrutiny about principle mechanisms which prednisolone fulfill its apoptotic properties through anti- and pro-apoptotic gene adjustment. Zhang et al., (2017) observed that prednisolone at dose of 2mg/kg/day in pediatric asthma cases who had good response could alter the pattern of methylation of OXT2, however; that was not seen in patient who had poor response. Moreover, in another study which was done on Trabeccular meshwork (TM) cells by Matsuda and colleagues demonstrated that treatment of these cells to the dexamethasone at dose of 100 nM could led to demethylation of CpG island on *MATN2* gene promotor which cause reduction in its gene expression (Matsuda et al., 2015). As well as, further study on effects of prednisolone on *MDR1* gene promotor methylation has approved the epigenetic potential of prednisolone in case of changing the gene expression profile of specific genes (Zadi Heydarabad et al., 2018). In this essay, the status of methylation on BAX and BCL2 promotor were evaluated in CCRF-CEM cells, following prednisolone treatment after 24 and 48 hours. Previously Kumar and colleagues showed which prednisolone could enhance the BAX/BCL2 ratio in some cancerous cells including CEM-C7H2 and THP1. This finding possibly suggests which pro-apoptotic effect of prednisolone could exert via intrinsic pathways (Kumar et al., 2006). BAX and BCL2 for having CpG island were considered as potential genes for influencing by the epigenetic phenomenon. Nevertheless, our MSP PCR data showed the same methylation status on BAX and BCL2 promoters in CCRF-CEM cells after and before prednisolone treatment. Furthermore, incubation time and different doses of prednisolone have no effect on DNA methylation of these two genes promotors in CCRF-CEM cells.

In conclusion, totally, the outcome of our study showed, prednisolone exerts its apoptotic effect through different pathways from promotor methylation of *BAX* and *BCL2* genes in CCRF-CEM cell line. However, the minor changes which are not detected via the MSP technique, are unavoidable. According to a reversible effect of the epigenetic phenomenon on gene regulation, more scrutiny about this intermediary event, in the case of cancer can promote the scientist to design a novel procedure to lower the side effect of therapies in leukemic patients.

## Abbreviation

Acute lymphoblastic leukemia (ALL); White Blood Cells (WBC); Signal transducer and activator of transcription 3 (STAT3); Nitric oxide (NO); Chronic myeloid leukemia (CML); Methylation specific PCR (MSP); Ubiquitin-like, containing PHD and RING finger domains, 1 (UHRF1).
